# Comorbidity Burden and Radiological Features Associated with *Legionella pneumophila* Pneumonia: A Retrospective Case–Control Study

**DOI:** 10.3390/pathogens15080866

**Published:** 2026-08-19

**Authors:** Nicola Serra, Teresa Maria Assunta Fasciana, Paola Di Carlo, Anna Giammanco, Antonio Cascio, Luca Pipitò, Dario De Luca, Caterina Carollo, Valentina Caputo, Martina Maria Federico, Consolato Maria Sergi, Emanuele Grassedonio, Tommaso Vincenzo Bartolotta

**Affiliations:** 1 Audiology Section, Department of Neuroscience, Reproductive Sciences and Dentistry, University of Naples Federico II, 80131 Naples, Italy; 2 Department of Health Promotion, Maternal-Childhood, Internal Medicine of Excellence G. D’Alessandro, University of Palermo, 90127 Palermo, Italy; teresa.fasciana@unipa.it (T.M.A.F.); antonio.cascio03@unipa.it (A.C.); luca.pipito@unipa.it (L.P.); valentina.caputo@unipa.it (V.C.); martinamaria.federico@you.unipa.it (M.M.F.); 3 Infectious and Tropical Disease Unit and Sicilian Regional Reference Center for the Fight Against AIDS, AOU Policlinico “P. Giaccone”, 90127 Palermo, Italy; 4 Legionella Reference Laboratory, University of Palermo, 90127 Palermo, Italy; anna.giammanco@unipa.it; 5 Spinal Unit at the P.O. “Villa delle Ginestre” of the ASP Palermo, Via Castellana, 145, 90135 Palermo, Italy; dario.deluca@asppalermo.org; 6 Section of Nephrology, Department of Health Promotion, Maternal-Childhood, Internal Medicine of Excellence G. D’Alessandro, University of Palermo, 90127 Palermo, Italy; caterina.carollo@unipa.it; 7 Department of Laboratory Medicine and Pathology, University of Alberta, Edmonton, AB T6G 2R3, Canada; sergi@ualberta.ca; 8 Section of Radiology, Department of Biomedicine, Neuroscience and Advanced Diagnostics (BiND), University of Palermo, 90127 Palermo, Italy; emanuele.grassedonio@unipa.it; 9 Section of Emergency Radiology Unit of Policlinico University Hospital “Paolo Giaccone”, Department of Biomedicine, Neuroscience and Advanced Diagnostics (BiND), University of Palermo, 90127 Palermo, Italy; tommasovincenzo.bartolotta@unipa.it

**Keywords:** *Legionella pneumophila*, community-acquired pneumonia, Legionnaires’ disease, comorbidity burden, Charlson Comorbidity Index, radiological findings

## Abstract

**Background**: *Legionella pneumophila* is a leading cause of community- and hospital-acquired pneumonia (HAP/CAP), although the clinical and radiological features associated with infection remain incompletely defined. This study aimed to identify clinical, comorbidity-related, and chest computed tomography (CT) features associated with *L. pneumophila* pneumonia in hospitalized adults with community-acquired pneumonia (CAP). **Methods**: A retrospective case–control study was conducted at a tertiary-care university hospital in Italy between January 2019 and August 2025. Eighty patients positive for *L. pneumophila* were compared with 79 controls negative for *L. pneumophila*. Demographic, clinical, microbiological, and radiological data were collected, and factors independently associated with infection were evaluated using multivariable logistic regression analysis. **Results**: Patients with *L. pneumophila* infection exhibited a greater comorbidity burden, particularly heart failure, and were more frequently smokers. Bilateral lung involvement and hilar/mediastinal lymphadenopathy were significantly more common among *Legionella*-positive patients, whereas overall pulmonary complications and bilateral pleural effusion were more frequently observed in controls. Multivariable analysis identified comorbidity burden as an independent positive predictor of *L. pneumophila* infection, while intensive care unit admission, oxygen therapy, and pulmonary complication score were independently associated with a lower likelihood of infection. **Conclusions**: The integration of comorbidity assessment with readily available chest CT findings may help raise clinical suspicion of *L. pneumophila* pneumonia and support consideration of appropriate diagnostic testing.

## 1. Introduction

*Legionella pneumophila* is an important cause of severe pneumonia acquired in both community and healthcare settings (CAP and HAP) and is associated with substantial morbidity and mortality, particularly among older adults, patients with chronic comorbidities, and immunocompromised individuals [1,2].

Although *L. pneumophila* accounts for the majority of reported cases of Legionnaires’ disease worldwide, other *Legionella* species may also cause clinically significant infection. In particular, *Legionella longbeachae* has emerged as an important human pathogen in specific geographic regions and exhibits epidemiological characteristics that differ from those of *L. pneumophila*. Nevertheless, *L. pneumophila* remains the predominant species responsible for Legionella infections in Europe and was the focus of the present investigation.

Although *Legionella* species account for only a minority of CAP cases, they represent one of the most important causes of atypical pneumonia requiring hospitalization and intensive care, making early recognition essential for appropriate antimicrobial treatment and improved clinical outcomes [2,3,4].

Over the past decade, the epidemiology of Legionnaires’ disease has changed considerably. Italy recently reported approximately 4627 cases of legionellosis, representing an 18–20% increase compared with the previous year [5]. This confirms Italy as the European country with the highest absolute number of reported cases. This trend is likely attributable to multiple factors, including an aging population, increased clinician awareness, wider use of microbiological diagnostic tests, and climate-related environmental changes that promote *Legionella* proliferation in natural and artificial water systems [6,7]. These epidemiological changes have renewed interest in strategies that facilitate earlier recognition of Legionella infection in patients presenting with CAP [3,4,8,9,10].

Despite important advances in laboratory diagnostics, the early diagnosis of *Legionella* pneumonia remains challenging. Urinary antigen testing and molecular assays have substantially improved diagnostic accuracy; however, these investigations are generally performed only after *Legionella* infection is clinically suspected [11,12]. Because the initial clinical presentation frequently overlaps with that of other bacterial pneumonias, delayed diagnostic suspicion remains one of the principal barriers to timely initiation of appropriate anti-*Legionella* therapy [11,13,14]. Consequently, the identification of readily available clinical and radiological features that can increase the pre-test probability of *Legionella* infection remains highly relevant in routine clinical practice [11,15].

*L. pneumophila* is a facultative intracellular microorganism that replicates within alveolar macrophages and monocytes, inducing a complex inflammatory response that frequently extends beyond the respiratory tract in pulmonary infections [16,17]. This distinctive pathophysiological behavior explains the multisystem manifestations that characterize Legionnaires’ disease and suggests that its thoracic imaging phenotype may also differ from that observed in conventional bacterial pneumonia. Previous computed tomography (CT) studies have described bilateral consolidations, ground-glass opacities, and multifocal pulmonary involvement in patients with *Legionella* pneumonia; however, the specificity and clinical utility of these findings remain incompletely defined, particularly in comparison with other causes of CAP [18,19,20].

In Italian emergency departments, chest CT is frequently performed during the initial evaluation of patients with severe community-acquired pneumonia because of their clinical presentation and the need to rapidly identify pulmonary complications. Consequently, radiological information is often available before microbiological confirmation. The integration of chest CT findings with clinical characteristics and host-related risk factors may provide additional clinical context when evaluating patients with community-acquired pneumonia and may contribute to clinical suspicion of *L. pneumophila* infection before microbiological confirmation [5,17,19,21].

In addition to imaging findings, host-related characteristics may play an important role in determining susceptibility to *Legionella* infection. Advanced age, smoking, chronic respiratory disease, cardiovascular disease, diabetes mellitus, malignancy, immunosuppression, and other chronic conditions have all been associated with an increased risk of Legionnaires’ disease [3,8,18,19,22,23]. Nevertheless, relatively few studies have evaluated whether overall comorbidity burden, beyond individual chronic conditions, contributes to the identification and clinical characterization of patients with *Legionella* pneumonia.

Therefore, the aim of the present study was to investigate the contribution of comorbidity burden and chest CT findings to the early recognition of *L. pneumophila* pneumonia among adults hospitalized with community-acquired pneumonia.

## 2. Materials and Methods

### 2.1. Study Design

This retrospective case-control study was conducted between January 2019 and August 2025 at the Policlinico University Hospital Paolo Giaccone (A.U.O.P.), University of Palermo, Italy, a tertiary-care academic teaching hospital designated as a second-level Emergency Department (DEA Level II). The institution serves as a major referral center for Western Sicily, has more than 500 hospital beds, and manages approximately 15,000 inpatient admissions and more than 50,000 Emergency Department visits annually [24]. The study was conducted in accordance with the Strengthening the Reporting of Observational Studies in Epidemiology (STROBE) statement [25]. The STROBE checklist and flowchart are provided in the Supplementary Materials (Table S1 and Figure S1, respectively).

Patients presenting with community-acquired pneumonia were initially evaluated in the Emergency Department and subsequently admitted to the most appropriate hospital ward according to disease severity, comorbidities, and bed availability. Clinical management was provided by a multidisciplinary team including emergency physicians, internal medicine physicians, pulmonologists, intensivists when required, and infectious disease specialists.

In this study, the patients were stratified into control and study groups (CG and SG, respectively). Patients aged ≥18 years who were hospitalized for community-acquired pneumonia (CAP) were classified according to microbiological testing for *Legionella pneumophila* performed within 48 h of emergency department admission.

CAP was defined according to the latest ATS/IDSA and ATS Clinical Practice Guidelines [26,27].

The control and study groups were composed of 79 and 80 hospitalized patients, respectively, who underwent urinary antigen testing, respiratory panels, and BioFire FilmArray™ PNplus Panels (BioFire Diagnostics, Salt Lake City, UT, USA), all performed within 48 h of admission [4].

To minimize selection bias, controls were selected among hospitalized patients with community-acquired pneumonia during the same study period and were chosen to achieve overall comparability with cases regarding age, sex, calendar year, season of admission, and sample size. No formal matching procedure was performed. Patients with HIV infection, SARS-CoV-2 infection, active tuberculosis, or hematological disorders were excluded. Patients who had received guideline-concordant anti-*Legionella* antimicrobial therapy for more than 2 days before presentation to the Emergency Department were excluded from the study [4,26,27].

All patients were admitted to the emergency radiology unit of A.U.O.P. and defined as having a new or progressive existing chest computed tomography infiltrate, in addition to a clinical manifestation suggesting a respiratory infection (fever and respiratory symptoms). A chest CT scan was performed according to Policlinico Universitario Hospital “Paolo Giaccone” guidelines [28,29]. Chest CT examinations were independently reviewed by experienced radiologists who were blinded to microbiological results.

Data on demographics, underlying diseases, pathogens, and ICU admission were retrospectively collected from the electronic records.

ICU admission and oxygen therapy variables referred to the initial management in the Emergency Department or emergency critical care area at presentation and not to events occurring later during hospitalization.

Chronic lung disease comorbidities included chronic obstructive pulmonary disease, interstitial lung disease, bronchiectasis, emphysema, asthma, and chronic bronchitis. Additionally, heart failure comorbidity included all patients, according to Bozkurt B. et al. [30]. The authors classify the medical setting in three categories—medicine, surgery, and intensive care unit (ICU)—reflecting differences in procedures and patient populations. Particularly, the medicine area included cardiology, endocrinology, gastroenterology, geriatrics, hematology, infectious diseases, internal medicine, nephrology, neurology, oncology, and pulmonology wards. The surgery area included cardiovascular surgery, general surgery, neurosurgery, oncological surgery, otolaryngology, orthopedic surgery, plastic surgery, urology, vascular surgery, and obstetrics and gynecology. Finally, the intensive care unit included general intensive care, postoperative care, and specialized care.

Urine samples were collected to determine the presence of *L. pneumophila Sg 1–15 L. longbeachae* antigens in urine by DiaSorin S.p.A. (Saluggia, Italy), as previously reported [4,31]. Bronchoalveolar Lavage (BAL)/mini-BAL/Protected Specimen Brush (PSB) samples were collected from patients hospitalized in the ICU and in other medical intensive care units and underwent microbiological investigation as previously reported [4,31,32].

Diagnostic testing was performed in accordance with current international recommendations for Legionella diagnosis [26,27]. Cases were defined as patients with microbiologically confirmed *L. pneumophila* infection based on a positive urinary antigen test and/or molecular detection of *L. pneumophila* in respiratory specimens using the BioFire FilmArray™ Pneumonia Panel Plus, (BioFire Diagnostics, Salt Lake City, UT, USA). Although diagnostic testing also allowed the evaluation of other clinically relevant *Legionella* species, including *L. longbeachae*, no cases attributable to non-*pneumophila Legionella* species were identified during the study period. Therefore, all Legionella-positive patients included in the present study had microbiologically confirmed *L. pneumophila* infection. We acknowledge that urinary antigen testing alone may have limited sensitivity and primarily detects *L. pneumophila* serogroup 1; therefore, molecular testing was also performed whenever respiratory specimens were available. Bacterial and fungal pathogens were identified using standard sputum culture and/or BioFire FilmArray Pneumonia Panel Plus, (BioFire Diagnostics, Salt Lake City, UT, USA), which can identify 15 typical bacteria, three atypical bacteria, and seven antimicrobial resistance (AMR) genes, including *Chlamydia pneumoniae*, *Mycoplasma pneumoniae*, and *L. pneumophila* [4,31,32].

Antibiotic therapy was initiated in the emergency department in accordance with the hospital guidelines [33].

### 2.2. Statistical Analysis

Categorical variables are expressed as absolute numbers and percentages, while continuous variables are reported as mean and standard deviation (SD) or median and interquartile intervals (IQRs), as appropriate.

Normality of continuous variables was assessed using the Shapiro–Wilk test. For comparisons between two groups, the unpaired *t*-test was used for normally distributed variables, while the Mann–Whitney U test was applied for non-normally distributed variables.

Differences in proportions between groups were evaluated using the chi-square test or Fisher’s exact test, as appropriate. When comparing more than two categories, post hoc analysis was performed using adjusted standardized residuals and Z-tests.

A multivariable logistic regression analysis was conducted to identify factors independently associated with *L. pneumophila* infection. Variables included in the model were selected based on statistical significance in univariate analysis (*p* < 0.05) and clinical relevance.

For this analysis, the following variables were considered dichotomous: Intensive Care Unit (ICU) admission (0 = no, 1 = yes), oxygen therapy (0 = no, 1 = yes), smoking status (0 = no, 1 = yes), and lung involvement (0 = unilateral, 1 = bilateral). ICU admission and oxygen therapy referred to patient status and management decisions made during the initial Emergency Department evaluation.

Comorbidities were quantified using the Charlson Comorbidity Index (CCI), a validated scoring system that assigns weighted points to specific conditions based on their impact on mortality risk [34,35]. The total CCI score for each patient was calculated as the sum of all weighted conditions. Higher scores indicate a greater comorbidity burden. In our study, the CCI was calculated as follows: liver cirrhosis (moderate–severe) = 3, dialysis = 2, diabetes = 1, heart failure = 1, dementia = 1, chronic lung diseases = 1, and hypertension = 0. Regarding lung complications, they included pneumothorax, cavitation, pulmonary embolism, hilar/mediastinal lymphadenopathy, unilateral pleural effusion, bilateral pleural effusion, and acute respiratory distress syndrome (ARDS).

As no validated scoring system is available for these conditions, a lung complication score was constructed based on clinical severity. Higher weights were assigned to conditions associated with greater respiratory impairment. The score was defined as follows: ARDS = 3; pneumothorax, pulmonary embolism, and bilateral pleural effusion = 2; cavitation, lymphadenopathy, and unilateral pleural effusion = 1.

In patients with multiple lung complications, the total score was calculated as the sum of all weighted conditions. This score was used for exploratory analysis. Patients with incomplete clinical, microbiological, or radiological data were excluded from the analysis.

To assess the adequacy of the sample size for multivariable modeling, the events-per-variable (EPV) approach was considered. The final logistic regression model included five covariates (ICU admission, oxygen therapy, smoking status, Charlson Comorbidity Index, and lung complication score) and was based on 80 patients with microbiologically confirmed *L. pneumophila* infection (events). Therefore, the ratio between events and covariates was 16 (EPV = 80/5 = 16), exceeding commonly recommended thresholds for logistic regression analyses and supporting the stability of the exploratory multivariable model [36,37].

All tests with a *p*-value < 0.05 were considered statistically significant. Statistical analysis was performed using MATLAB (Matrix Laboratory) analytical toolbox R2008b (MathWorks, Natick, MA, USA).

## 3. Results

The control group (CG) was composed of 72.2% (57) males and 27.8% (22) females, with ages ranging from 25 to 94 years, a mean of 65.2 years, and a standard deviation of 17.2 years. The study group (SG) comprised 66.3% (53) males and 33.8% (27) females, with ages ranging from 26 to 95 years, a mean of 67.2 years, and a standard deviation of 15.8 years. Among Legionella-negative controls, the microbiological investigations identified a heterogeneous spectrum of pathogens. The most frequently detected Gram-negative bacteria included *Pseudomonas aeruginosa*, *Haemophilus influenzae*, *Klebsiella pneumoniae*, and *Moraxella catarrhalis*, whereas Gram-positive isolates included *Streptococcus pneumoniae* and *Staphylococcus aureus*. Respiratory viruses were also identified, predominantly influenza A virus, parainfluenza viruses, and human rhinovirus/enterovirus. In addition, a small number of cases of *Mycoplasma pneumoniae* infection were detected during the study period.

Table 1 summarizes the demographic, clinical, microbiological, and comorbidity-related characteristics of both groups.

Table 1 shows significant differences between the control group (CG) and the study group (SG) in ICU admission, oxygen therapy, smoking status, and comorbidity-related characteristics.

ICU admission and oxygen therapy were more frequently observed in the CG than in the SG (79.7% vs. 63.8%, *p* = 0.0256, and 78.5% vs. 63.8%, *p* = 0.0412, respectively).

Conversely, smoking was more prevalent in the SG than in the CG (83.8% vs. 67.1%, *p* = 0.0149). Regarding underlying conditions, patients with *L. pneumophila* infection exhibited a greater burden of comorbidities, with heart failure being the condition most strongly represented in the SG (41.3%, *p* = 0.0004).

Table 2 summarizes the radiological findings observed during the acute phase of *L. pneumophila* pneumonia. Lung complications included pneumothorax, cavitation, pulmonary embolism, hilar and/or mediastinal lymphadenopathy, unilateral pleural effusion, bilateral pleural effusion, and acute respiratory distress syndrome (ARDS).

Table 2 shows that bilateral lung involvement was more frequent in the SG than in the CG (77.5% vs. 62.0%, *p* = 0.0336). Overall pulmonary complications were more frequently observed in the CG than in the SG (83.5% vs. 62.5%, *p* = 0.0029), although the distribution of specific radiological abnormalities differed between groups. Analysis of individual abnormalities revealed significant differences in bilateral pleural effusion and hilar/mediastinal lymphadenopathy.

Variables significantly associated with *L. pneumophila* infection in univariate analyses were entered into a multivariable logistic regression model (Table 3). The final model included five covariates (ICU admission, oxygen therapy, smoking status, Charlson Comorbidity Index, and lung complication score) and was based on 80 microbiologically confirmed *L. pneumophila* cases (events). The adequacy of the sample size for multivariable modeling was assessed using the events-per-variable (EPV) criterion. The EPV was calculated as the number of outcome events divided by the number of covariates included in the model (EPV = E/*nC*), where *E* represents the number of events and *nC* the number of covariates. Accordingly, the present model yielded an EPV of 16 (EPV = 80/5 = 16), exceeding the commonly recommended minimum threshold of 10 events per variable and supporting the stability of the exploratory multivariable analysis [36,37].

The multivariable logistic regression model was statistically significant (*p* < 0.0001), indicating that the included variables were collectively associated with *L. pneumophila* infection.

ICU admission, oxygen therapy, and lung complication score were independently associated with a lower probability of *L. pneumophila* infection (OR = 0.17, *p* = 0.0006; OR = 0.28, *p* = 0.0047; OR = 0.57, *p* = 0.0078, respectively).

Conversely, comorbidity burden, assessed by the Charlson Comorbidity Index (CCI), was identified as an independent positive predictor, with each unit increase associated with a 61% higher likelihood of infection (OR = 1.61, *p* = 0.0008).

Smoking status showed a positive trend but did not reach statistical significance (OR = 2.12, *p* = 0.09).

**Figure 1:**
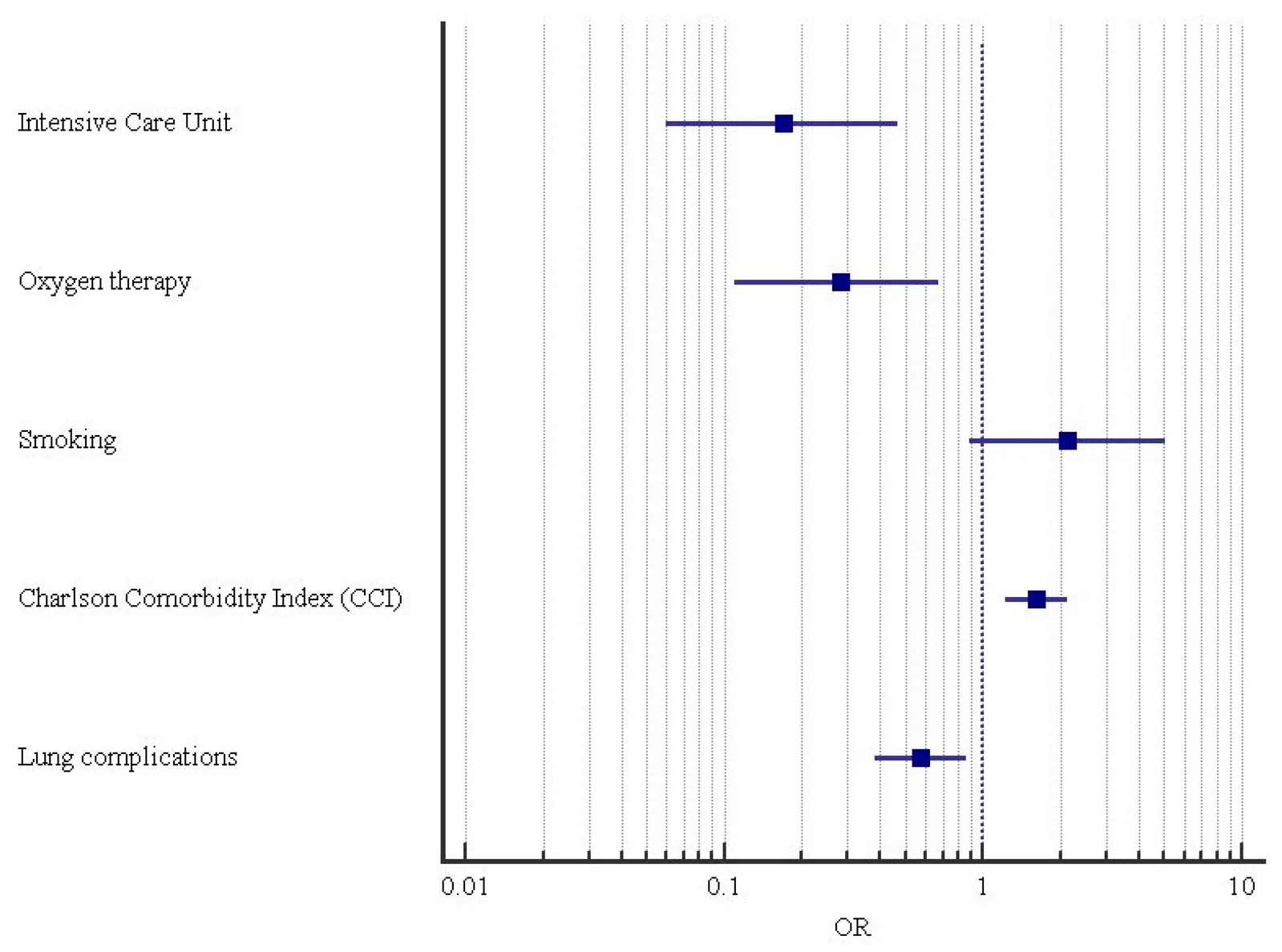
Forest plot of multivariable logistic regression analysis identifying factors associated with *Legionella pneumophila* infection.

The odds ratios and corresponding 95% confidence intervals derived from the multivariable logistic regression model are presented in Figure 1.

Figure 1 shows the magnitude and direction of the associations identified in the multivariable model. The Charlson Comorbidity Index (CCI) was the only independent factor positively associated with *L. pneumophila* infection, whereas ICU admission, oxygen therapy, and lung complication score showed significant inverse associations. Smoking did not retain statistical significance.

## 4. Discussion

The rationale for the present study stems from the growing epidemiological relevance of Legionnaires’ disease in Italy, where incidence has increased in recent years. This trend likely reflects the combined effects of climate change, population aging, increased environmental exposure to *Legionella* spp., greater clinical awareness, and the more widespread use of molecular diagnostic techniques that improve the detection of Legionella infections. Warmer temperatures and altered rainfall patterns may promote bacterial proliferation in both natural and artificial water systems, raising concerns that the burden of infection will continue to increase [2,3,4].

In Italian emergency departments, patients presenting with severe community-acquired pneumonia (CAP) routinely undergo chest computed tomography (CT) due to clinical severity. This widespread use of thoracic imaging provides an opportunity to identify radiological patterns that, although not specific, may increase clinical suspicion for *Legionella* pneumonia at presentation. Accordingly, we investigated whether host characteristics, comorbidity burden, and imaging findings were associated with *L. pneumophila* infection and could help increase clinical suspicion in patients presenting with community-acquired pneumonia, supporting timely microbiological testing and appropriate antimicrobial therapy [8,20,38].

The term ‘atypical pneumonia’ has historically been applied to pathogens such as *Legionella pneumophila*, *Mycoplasma pneumoniae*, and *Chlamydia pneumoniae* because of their microbiological characteristics and the difficulty of detection using conventional diagnostic methods. Our findings do not seek to redefine this classification. Rather, they suggest that certain host-related and radiological features may increase clinical suspicion of *L. pneumophila* infection among patients presenting with community-acquired pneumonia.

The Charlson Comorbidity Index (CCI) was independently associated with *L. pneumophila* infection, supporting the hypothesis that cumulative comorbidity burden may contribute to host susceptibility to *Legionella* pneumonia, particularly among older adults [39,40,41].

Another important finding of our study concerns the radiological phenotype of *Legionella pneumophila* pneumonia. Patients with *Legionella* infection more frequently exhibited bilateral pulmonary involvement and hilar or mediastinal lymphadenopathy than patients with non-*Legionella* community-acquired pneumonia. Our findings are consistent with previous studies describing bilateral pulmonary involvement and hilar or mediastinal lymphadenopathy among patients with *Legionella* pneumonia. Previous CT studies have shown that initially focal infiltrates may extend to adjacent lobes and become bilateral, even despite appropriate antimicrobial therapy and clinical improvement [17,42,43,44]. In contrast, Rémi Poirier et al. reported that radiographic and tomographic manifestations of LD are nonspecific and like those found in community-acquired pneumonia of other bacterial origin [45]. The distinctive pathogenic mechanism of the higher frequency of bilateral pulmonary involvement, as well as the significantly greater prevalence of hilar and mediastinal lymphadenopathy observed in our cohort, may be due to the higher prevalence of heart failure in our enrolled population [46,47]. As reported by Di Carlo et al., heart failure was more frequent among *Legionella* cases and may increase susceptibility to *L. pneumophila* infection [4]. Therefore, the interpretation of mediastinal lymphadenopathy as a radiological feature associated with *Legionella* pneumonia should be considered with caution, given the potential confounding effect of heart failure.

Conversely, pulmonary complications were more frequently observed in the control group. This finding suggests that radiological complexity alone may not be a distinguishing feature of *Legionella* pneumonia. Rather, the higher frequency of complications in controls may reflect differences in disease severity, underlying clinical characteristics, or etiological heterogeneity between the study groups [32,48]. Furthermore, pathogens known to cause pulmonary disease, as well as other opportunistic pulmonary microorganisms, are associated with seasonality in our geographical area [49,50,51,52]. In summary, this analysis suggests that comorbidity burden may represent a major factor associated with *L. pneumophila* infection in patients with community-acquired pneumonia. Acute clinical severity and radiological complexity played a lesser role in risk stratification. These data suggest that a careful assessment of chronic conditions should guide clinical suspicion and subsequent diagnostic decisions, thereby optimizing the management of this population. Importantly, ICU admission and oxygen therapy in this study referred to clinical status and management decisions made during the initial Emergency Department evaluation rather than to events occurring later during hospitalization. Therefore, their inverse association with *L. pneumophila* infection reflects differences observed at presentation and should not be interpreted as evidence of a causal relationship.

Moreover, our findings support the hypothesis that bilateral pulmonary involvement and hilar or mediastinal lymphadenopathy, when interpreted together with a higher burden of comorbidities, define a clinical profile associated with *L. pneumophila* infection. These readily available clinical and imaging features may contribute to clinical suspicion of *L. pneumophila* infection and support consideration of appropriate microbiological testing when clinically indicated. Rather than replacing microbiological confirmation, their integration into routine clinical assessment may support earlier diagnostic investigation for *L. pneumophila* infection and consequently contribute to reducing diagnostic delay, particularly in countries such as Italy, where the incidence of Legionnaires’ disease is increasing [6,32,52,53].

### Limitations

This study has several limitations that should be considered when interpreting the results.

First, the retrospective design may introduce selection bias and limits the ability to establish causal relationships between the identified predictors and *L. pneumophila* infection.

Second, the study was conducted at a single center, which may limit the generalizability of the findings to other settings or populations. Furthermore, the study was conducted in a tertiary-care university hospital and primarily involved patients evaluated in an emergency-care setting. Therefore, the findings may not be fully generalizable to lower-risk populations, primary care settings, or healthcare systems characterized by different referral pathways and access to specialized care. However, studies specifically investigating *L. pneumophila* pneumonia during the initial emergency assessment remain relatively limited, and this context should be considered when interpreting the present findings.

Third, although comorbidities were quantified using the Charlson Comorbidity Index (CCI), an adapted approach was used to include multiple conditions within the same category, which may differ from the standard Charlson methodology. While this approach better reflects the overall burden of disease, it may reduce comparability with other studies.

Fourth, the lung complication score was not based on a validated scoring system but was instead derived from clinical severity. Although this approach allowed a more comprehensive assessment of pulmonary involvement, it should be considered exploratory and requires external validation.

Finally, although the study included a relatively large series of microbiologically confirmed *L. pneumophila* cases compared with previous single-center studies, the overall sample size remained relatively small. This may have limited statistical power, increased the possibility of type II error, and reduced the generalizability of the findings to other populations and healthcare settings. Nevertheless, the number of events available for the multivariable analysis was considered adequate in relation to the number of covariates included in the final model. Therefore, the present findings should be interpreted as exploratory and hypothesis-generating and require confirmation in larger multicenter cohorts.

## 5. Conclusions

In conclusion, this study shows that *L. pneumophila* community-acquired pneumonia is associated with a characteristic clinical and radiological profile compared with other causes of CAP. A higher comorbidity burden, particularly as assessed by the Charlson Comorbidity Index, emerged as the primary independent factor associated with infection, highlighting the importance of host susceptibility in the pathogenesis of *Legionella* disease. On chest CT, bilateral pulmonary involvement and hilar or mediastinal lymphadenopathy were more frequently observed in patients with *L. pneumophila* infection and may help raise diagnostic suspicion when interpreted within the appropriate clinical context. The integration of comorbidity assessment with readily available radiological findings may contribute to raising clinical suspicion of *L. pneumophila* infection and may support consideration of Legionella-specific diagnostic testing in appropriate clinical settings. However, these findings should be interpreted as associations observed within a retrospective case-control study rather than as validated diagnostic predictors and therefore require prospective confirmation before any clinical implementation.

Future multicenter prospective studies are warranted to validate these findings and assess their applicability in broader populations of patients with community-acquired pneumonia.

Future prospective studies may further investigate whether the clinical and radiological factors identified in this study have utility in broader clinical settings and whether their potential integration into clinical decision-support tools warrants evaluation. However, any such application would require rigorous prospective validation before implementation in clinical practice [54,55].

## Figures and Tables

**Table 1:** Comparison between negative (control group) and positive patients for *L. pneumophila* (SG).

Parameters	CG (Control Group)	SG (*L. pneumophila* Group)	CG vs. SG *p*-Value (Test)
*Patients*	79	80	
*Age*			
Mean (SD)	65.2 (17.2)	67.2 (15.8)	
Median (IQR)	70 (52, 79)	70 (58.5, 79)	0.5 (MW)
*Gender*			
Males	72.2% (57)	66.3% (53)	
Females	27.8% (22)	33.8% (27)	0.42 (C)
*Hospitalization* (days)			
Mean (SD)	16.1 (13.6)	20.4 (17.0)	
Median (IQR)	12 (8.0, 20.75)	14 (9.0, 28.0)	0.12 (MW)
*Intensive Care Unit (ICU)*	79.7% (63)	63.8% (51)	0.0256 * (C)
*Oxygen therapy*	78.5% (62)	63.8% (51)	0.0412 * (C)
*Non-invasive mechanical ventilation*	60.8% (48)	57.5% (46)	0.68 (C)
*Mortality*	30.4% (24)	21.2 (17)	0.19 (C)
*Smoking*	67.1% (53)	83.8% (67)	0.0149 * (C)
*Patients with comorbidities*	82.3% (65)	86.3% (69)	0.49 (C)
*Comorbidities type* †			
Diabetes	15.2% (12)	26.3% (21)	0.0047 * (F)
Heart failure (HF)	6.3% (5)	41.3% (33) **	
Chronic lung diseases	58.2% (46)	62.5% (50)	SG-HF: 41.3%, *p* = 0.0004 * (Z)
Hypertension	44.3% (35)	46.3% (37)	
Dialysis	10.1% (8)	26.3% (21)	
Liver cirrhosis	3.8% (3)	3.8% (3)	
Dementia	16.5% (13)	33.8% (27)	
*Sepsis*	19.0% (15)	27.5% (22)	0.21 (C)
*Cancer*	31.6% (25)	33.8% (27)	0.59 (C)
*Isolates* ††			
Gram−	19.0% (15)	31.3% (25)	
Gram+	8.9% (7)	10.0% (8)	0.11 (C)
Virus	13.9% (11)	7.5% (6)	

Note: † = some patients had more comorbidities; †† = some patients had more isolates; CG: negative for *L. pneumophila*; SG: positive for *L. pneumophila*; * = significant test; MW = Mann–Whitney test; C = chi-square test; F = Fisher’s exact test; ** = more frequent modality; Z = post hoc Z-test. Italicized terms indicate study variables; non-italicized terms indicate the corresponding categories, levels, or values.

**Table 2:** Radiological pulmonary findings of patients with *L. pneumophila*.

Parameters	CG (Negative for *L. pneumophila*)	SG (Positive for *L. pneumophila*)	CG vs. SG *p*-Value (Test)
*Lung involvement*			0.0336 * (C)
Unilateral	38.0% (30)	22.5% (18)	
Bilateral	62.0% (49)	77.5% (62)	
*Patients with lung complications*	83.5% (66)	62.5% (50)	0.0029 * (C)
*Lung complications type* †			0.0008 * (F)
Unilateral effusion	26.6% (21)	13.8% (11)	
Bilateral effusion (be)	21.5% (17) **	5.0% (4)	CG-be, 21.5%, *p* = 0.0137 * (Z)
Cavitation	5.1% (4)	2.5% (2)	
Pneumothorax	1.3% (1)	1.3% (1)	
ARDS ***	16.5% (13)	21.3% (17)	
Pulmonary embolism	8.9% (7)	1.3% (1)	
Hilar and/or mediastinal lymphadenopathy > 10 mm (lin)	5.1% (4)	18.8% (15) **	SG-lin, 18.8%, *p* = 0.0006 * (Z)
*Radiological findings*			
Interstitial pneumoniae	16.5% (13)	15.0% (12)	
Lobar pneumoniae	20.3% (16)	20.0% (16)	0.65 (C)
Interstitial plus lobar pneumoniae	20.3% (16)	13.8% (11)	
Bronchopneumonia or lobular pneumonia	43.0% (34)	51.3% (41)	

Note: *** = Acute respiratory distress syndrome; † = some patients had more *complications*; C = chi-square test; F = Fisher’s exact test; * = significant test; ** = more frequent modality; Z = post hoc Z-test. Italicized terms indicate study variables; non-italicized terms indicate the corresponding categories, levels, or values.

**Table 3:** Multiple logistic regression analysis among *Legionella_var* and significant variables described in Table 1 and Table 2.

Logistic Regression	Coefficient	Standard Error	OR	CI at 95%	*p*-Value
Null model vs. full model					<0.0001 * (C)
*Legionella_var*/*Intensive Care Unit*	−1.77	0.51	0.17	(0.06; 0.47)	0.0006 *
*Legionella_var*/*Oxygen therapy*	−1.29	0.46	0.28	(0.11; 0.67)	0.0047 *
*Legionella_var*/*Smoking*	0.75	0.44	2.12	(0.89; 5.03)	0.09
*Legionella_var*/*Charlson Comorbidity Index (CCI)*	0.48	0.14	1.61	(1.22; 2.12)	0.0008 *
*Legionella_var*/*Lung complication score*	−0.56	0.21	0.57	(0.38; 0.86)	0.0078 *
*Constant*	1.54	0.76	—	—	0.0431 *

Note: * = significant test; OR = odds ratios; CI = odds ratios confidence interval at 95%. The null model = −2ln(L_0_), where L_0_ was the likelihood of obtaining the observations if the independent variables did not affect the outcome; the full model: −2ln(L_0_), where L_0_ was the likelihood of obtaining the observations with all independent variables incorporated in the model; C = chi-square test. Italicized terms indicate variables formalized as numerical variables for statistical analyses.

## Data Availability

The research data were available from Paola Di Carlo and Tommaso Vincenzo Bartolotta.

## Supplementary Materials

The following supporting information can be downloaded at: https://www.mdpi.com/article/10.3390/pathogens15080866/s1, Figure S1: Study flowchart; Table S1: STROBE checklist.

## Abbreviations

The following abbreviations are used in this manuscript:

CT	Computer Tomography
ICU	Intensive Care Unit
HIV	Human Immunodeficiency Virus
CAP	Community-Acquired Pneumonia
CG	Control Group
SG	Study Group
