# Supplementary material for: Comorbidity Burden and Radiological Features Associated with Legionella pneumophila Pneumonia: A Retrospective Case–Control Study
Source: Pathogens. 2026 Aug 19;15(8):866. doi: 10.3390/pathogens15080866 (PMC13516180; doi:10.3390/pathogens15080866)
Supplement: Supplementary file 1 [file pathogens-15-00866-s001.zip › Figure S1.pdf]

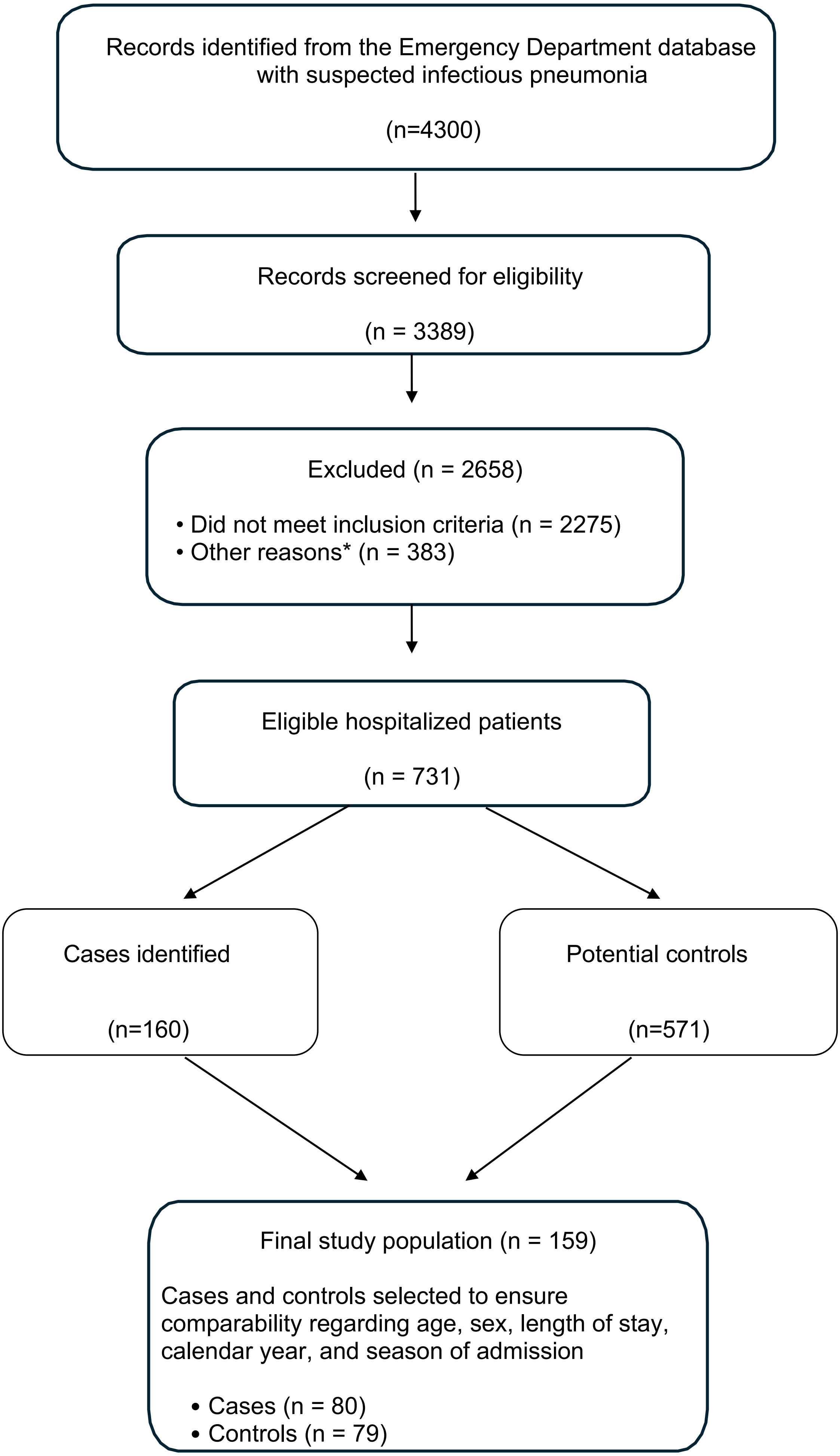

* Other reasons: patients who chose to receive care in private healthcare facilities or through their general practitioner.
